# SIGULS: Signal generator for user-specified laboratory signals

**DOI:** 10.1016/j.ohx.2026.e00750

**Published:** 2026-02-17

**Authors:** Abelardo Pérez Paz, Oliver Maximilian Zobel, Daniel J. Rixen

**Affiliations:** Chair of Applied Mechanics, TUM School of Engineering and Design, Technical University of Munich, Boltzmannstr. 15, 85748 Garching, Germany

**Keywords:** Open-source, Signal generation, Experimental dynamics, Acquisition hardware, Measurement equipment, Vibration analysis

## Abstract

SIGULS (signal generator for user-specified laboratory signals) is an open-source excitation signal generator designed for use in structural dynamics testing. It offers the user full control over the shape of the excitation signal, is comparatively affordable, and can be operated independently from any data acquisition system being used. It is based on the ESP32-S3 microcontroller and the AD5781 Digital-to-Analog Converter. This publication presents the characteristics of the device and lays out some of the key design decisions. A structural dynamics experiment is carried out to evaluate the performance and utility of the device compared to commercial alternatives. The comparatively high cost and limited flexibility of the available alternatives motivates the development of this device.

## Graphical abstract

**Figure:**
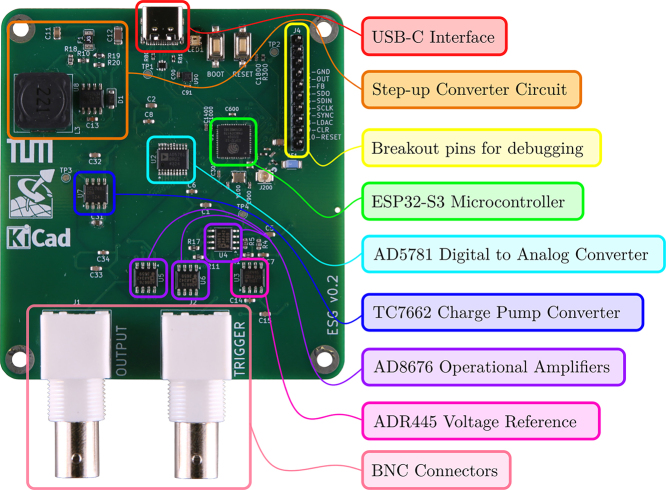

## Specifications table

**Table:** 

**Hardware name**	SIGULS
**Subject area**	Educational tools and open source alternatives to existing infrastructure
**Hardware type**	Measuring physical properties and in-lab sensors
**Closest commercial analog**	No direct commercial analog is available.
**Open source license**	CC-BY 4.0 (Hardware)/MIT (Software)
**Cost of hardware**	≈$180/150 €
**Source file repository**	https://doi.org/10.5281/zenodo.17400147
**OSHWA certification UID**	DE000160

## 1 Hardware in context

**Fig. 1:**
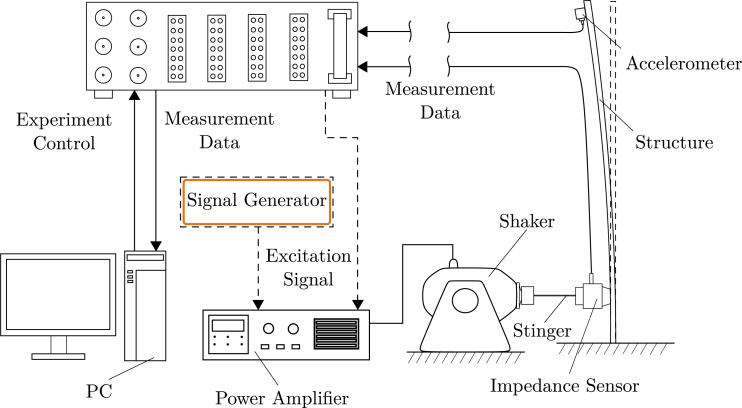
The schematic for the setup of a typical structural dynamics experiment.

SIGULS is an excitation signal generator tailored for structural dynamics testing. Structural dynamics tests require a method to excite the structure in a controlled way, as well as a method to capture the response of the structure. Excitation is most commonly carried out using impulse hammers or shakers. Where impulse hammers can produce good results and simplify the experimental setup, they offer only one possible form of excitation: an impulse. Shakers, on the other hand, can impose a variety of excitation types on the structure, for instance, making it possible to investigate only a certain frequency range of interest, e.g., using a swept sine. Shakers are controlled by supplying them with a power-amplified analog signal, the excitation signal. The device that generates this signal is often integrated with a data acquisition system (DAQ). Because the excitation signal does not carry the power needed to drive the shaker, it passes through a power amplifier on its way to the shaker. An exemplary experimental setup for structural dynamics tests is shown schematically in Fig. 1 [1].

The presented device offers the possibility to generate an analog signal of any shape up to a sampling rate of 100 kHz. Most signal generators (more commonly referred to as function generators) are meant for use in electronics, where the relevant frequency range spans up to the order of MHz [2]. In structural dynamics experiments, however, a frequency range in the order of 10 kHz is usually of interest [3]. Therefore, the random signals generated by conventional function generators include frequency content that is not useful for structural dynamics testing. To use random signals from these function generators, they need to be bandlimited using an external low-pass filter. Some of the signal types typically used in structural dynamics testing, like pseudo-random, burst-random, or windowed signals are not offered by conventional function generators [4]. The few signal generators that offer these options tend to be expensive. This is also the case for data acquisition systems with integrated excitation signal generators, which are expensive both in terms of the hardware itself, but also because of the software licenses required to operate them. SIGULS integrates a selection of common excitation signals for structural dynamics into its graphical user interface (GUI) and allows the user to choose any other tool (e.g., Python, Matlab, Julia, etc.) to design their signals with, as long as they can be exported to a CSV format, to be uploaded to the device.

Broadly, SIGULS can support laboratory tasks in the following ways:

• Providing a low-cost device tailored for excitation signal generation in structural dynamics testing.
• Offering suitability for use in mobile experiments or test nodes outside traditional laboratory environments thanks to a small form factor and low power requirements.
• Providing an open-source, transparent alternative to black-box laboratory equipment for education and flexible development of experimental test design.
• Allowing for full customizability of software features (e.g., WiFi connectivity) and hardware features (e.g., external RAM to expand memory for signal samples), as needed in the respective field of research.

## 2 Hardware description

This section will cover the electronic design of SIGULS as well as the design of the accompanying software. SIGULS uses a 4-layer PCB to ensure good signal quality. The intended layer stack-up is: top signal plane, power plane, ground plane, and bottom signal plane.

### 2.1 Circuit design

**Fig. 2:**
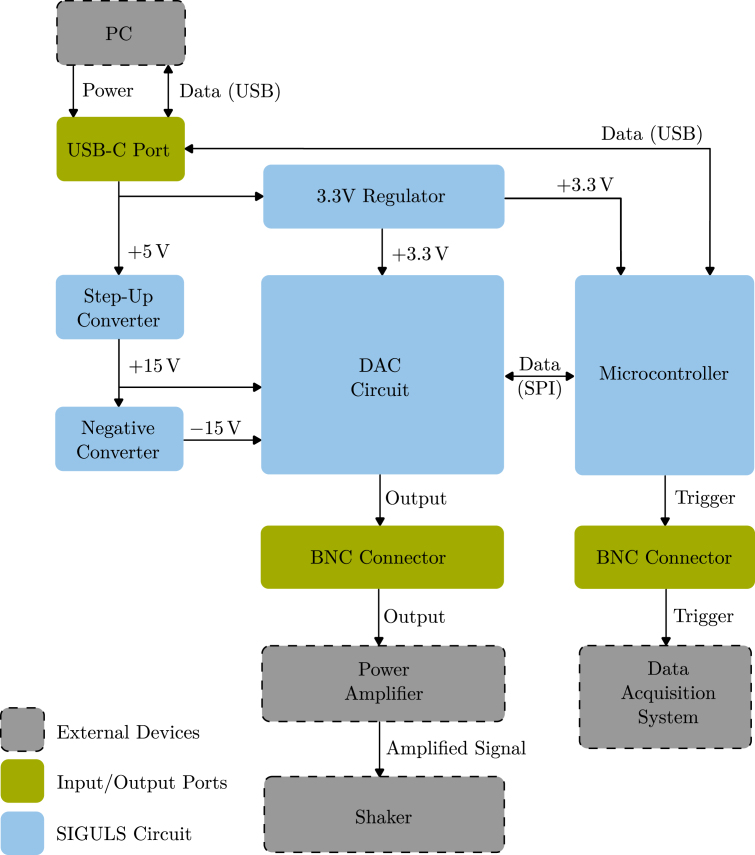
Schematic of the circuit’s modules.

The various modules within the SIGULS circuit are visualized in Fig. 2. SIGULS is built around the *Analog Devices AD5781* DAC with 18-bit resolution and a 35 MHz Serial Peripheral Interface (SPI). This DAC was selected for its high accuracy, low noise and low temperature drift. Conveniently, a reference circuit has already been designed around the *AD5781* by *Analog Devices* [5]. The design of SIGULS is based on the *CN0177* reference circuit [6].

The DAC requires precise 10 V and -10 V reference voltages to generate its ±10 V output signal accurately. Apart from resistors and capacitors, an *ADR445* 5 V voltage reference and three *AD8676* operational amplifiers are used for this purpose. The latter require a ±15 V, and the implementation of multiple other sub-circuits. An in-depth experimental evaluation of the signal quality from the output of the AD5781 is provided in its datasheet and will thus be omitted in our work. Although the performance of the DAC can be influenced by the rest of the board, we choose to omit further evaluation or calibration of the output signal, because precision at this level is not required for structural dynamics testing. Because the signal passes through an amplifier, where often the exact gain is not known, then through a shaker, which has its own dynamics, and then creates a force on the structure which also interacts with this force, the force signal will already be different compared to the electrical signal generated. Additionally, the excitation force is almost always measured and this measurement is relevant for the structural dynamics test. The only important characteristic of the generated signal is its frequency content, which is validated in Section 7 [5].

An *ESP32-S3* microcontroller is used to control the DAC. Since this application mainly requires an SPI interface which is ubiquitous across microcontrollers, almost any modern microcontroller could have been a good choice. However, the chip offers WiFi connectivity, which could be a practical feature on the device in the future. Usually, their comparatively high power usage is listed as a drawback of the *ESP32* family of boards. Because minimizing power usage is not a concern in this application this drawback can be neglected. We will use this microcontroller in combination with *Arduino-ESP32 Core v2.0.17* from git commit hash *dcc1105b* for the firmware [7].

A USB-C port is used to power the board, flash the microcontroller, and communicate with the microcontroller via the native USB interface on the *ESP32-S3* microcontroller while the program is running. The D+ and D− data lines from the USB-C interface are used to establish communication between the board and a PC, the USB-C port’s 5 V supply powers the board. Though the USB-C port supplies 5 V, the microcontroller and the DAC both also require a 3.3 V source. Therefore, the TexasInstrumentsTLV767 fixed 3.3 V positive electrode is used to regulate the 5 V down to 3.3 V.

A step-up converter circuit is used to generate the 15 V required by the DAC circuit from the 5 V supplied by the USB-C port. The central component in this circuit is the *MC34063* switching regulator by *Texas Instruments*. To operate this component as a step-up converter it has to be arranged together with a few passive components. In order to attain the -15 V source, the 15 V source is inverted using the *Texas Instruments TC7662* Charge Pump DC-to-DC Converter as a negative converter.

**Fig. 3:**
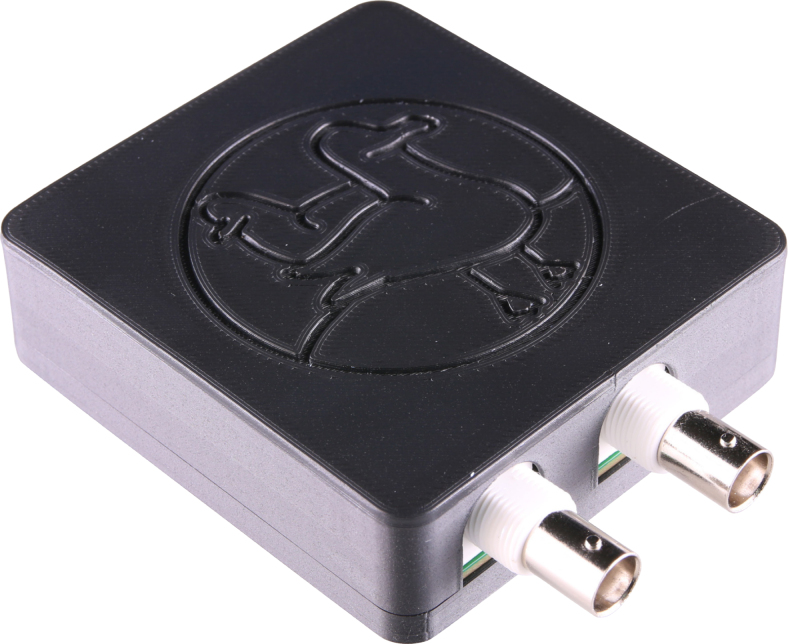
The SIGULS device.

**Fig. 4:**
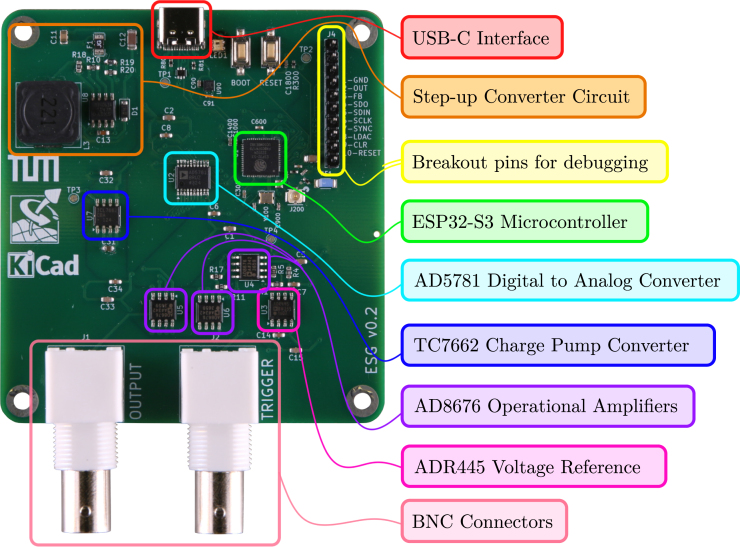
The SIGULS PCB with labels on the most important components and sub-circuits.

To connect the board’s outputs to external devices, Bayonet Neill Concelman (BNC) coaxial connectors are used. The board has two BNC connectors, one for the excitation signal and one for a trigger signal. This second signal may be connected to the data acquisition system (DAQ) during the experiment to trigger the start of the measurement at the same time as the output of the excitation signal begins. When using the same excitation signal for multiple measurements, the trigger signal helps to synchronize them. For instance, this can be of use when averaging over a series of measurements. An LED on SIGULS will light up blue if the board is connected, and green if the board is generating an output signal. The fully assembled device can be seen in Fig. 3. A labeled view of the PCB is shown in Fig. 4.

### 2.2 Software description

In this chapter, the software that controls the excitation signal generator will be described. The software has to solve four fundamental problems to make SIGULS function:

1. Generate the sequence of values that make up the desired excitation signal (see Section 2.2.1)
2. Provide a way to upload the signal data to the device (see Section 2.2.2)
3. Allow the user to start and stop the output of the excitation signal (see Section 2.2.3)
4. Establish fast communication between the microcontroller and the DAC (see Section 2.2.4)

#### 2.2.1 Generating data for excitation signals

The analog output signal from SIGULS is generated by sending discrete values to the DAC via SPI and subsequently triggering the update of the DAC’s output signal. Before this can take place, a sequence of values needs to be made available in some way.

SIGULS outsources the generation of signals to the user’s PC. On a PC, software like Python or MATLAB can be used to generate arrays of sampled values from a given waveform chosen by the user. Remarkably, the PyExSi Python library [8] offers this functionality specifically for excitation signals used in structural dynamics testing. Though this library is used to design signals in the SIGULS GUI, any tool the user likes can be used to design signals for SIGULS as long as it can export the samples to a CSV file, consisting of a single row that contains all the samples. In this subsection, the design of bandlimited pseudo-random signals as implemented in the SIGULS GUI will be explained as an example.

Using *PyExSi’s* pseudo_random() function, we can generate a random sequence of values. In theory, this signal would excite all frequencies as it is composed of a series of step signals. Band-limiting the random signals opens up the possibility to excite a specific region of interest within the frequency spectrum during an experiment. In our case, band-limiting is done using a fourth-order butterworth filter with an adjustable cut-off frequency.

Because the values have to be transferred to the DAC in an 18-bit format, the data generated using PyExSi needs to be mapped to this format. To apply the same formula for the mapping of any set of data generated, the data set is first normalized to a range between +1 and −1. To normalize the data, the entire array can simply be divided by the largest absolute value in the array.

Because of the limited amount of data that can be loaded onto SIGULS (discussed later in this chapter) the data must be looped over during longer experiments. To ensure there are no jumps in the signal when it starts over from the beginning, a tukey window is applied, which smoothly tapers the signal towards 0 at the beginning and end. Only the random signals are (optionally) windowed in this way, since swept sines are generated by PyExSi to contain a round number of periods, meaning there are no jumps in the signal if it is looped over.

For most experiments, one would ideally use the entire voltage range the DAC can offer to maximize resolution, and then adjust the gain on the power amplifier to set the amplitude of the excitation. The *AD5781* DAC takes 18 bits for the value and an additional bit for the sign. Setting the total of 19 bits all to one (0x7FFFF in hexadecimal) would result in the largest possible positive value (the positive reference voltage $V_{\text{REFP}}$), which is 10 V in our case. Inversely, setting all bits to zero would result in the largest possible negative value (the negative reference voltage $V_{\text{REFN}}$). To convert the values d we normalized to ±1 before to a scale between 0 and 1, we can use the expression $\frac{d+1}{2}$. Multiplying the range of possible values by the result of this expression and adding the minimum value effectively maps the normalized array we started out with to the value v on the 18-bit scale of the DAC, as can be seen in Eq. (1):

(1) $$\text{v}=\text{int}\left(\frac{d+1}{2}\cdot \left(\text{max}-\text{min}\right)+\text{min}\right)$$

where min=0x00000 and max=0x7FFFF for a ±10 V output [5].

The array of data is saved as a CSV file titled “data.csv” using Python’s CSV module. The CSV file contains the data as a single row.

#### 2.2.2 Loading data onto siguls

The samples from the user’s PC are loaded onto a file system on the microcontroller to be accessed during runtime. This minimizes transmission errors (by using existing flash tools) and loads the data directly into non-volatile memory, protecting it from power disconnection or resets of the microcontroller.

*ESP32* microcontrollers support three file systems: FATFS, SPIFFS and LittleFS. LittleFS stands out for its low resource occupancy and its efficiency in read, write and erase processes, and was therefore chosen as the file system for SIGULS. Uploading files to the microcontroller is done through a Python function. This function generates a binary image from the previously generated data.csv file using a mklittlefs command, and then executes an esptool command to flash the binary image onto the assigned region in the microcontroller’s memory. On the microcontroller, the CSV is stored in the form of raw bytes, with no additional CSV formatting. Generating and uploading an additional file called parameters.csv allows us to specify settings for SIGULS. For now, this has been implemented to set the output sampling rate on SIGULS [9].

Because reading from flash memory is slow compared to reading from RAM, the data is transferred from the file system into an array saved in RAM before starting the cyclical process of writing values to the DAC, in the interest of keeping sampling rates as high as possible. Since the available RAM is much smaller than the amount of non-volatile memory that can be allocated to LittleFS files, the length of signals generated by SIGULS is limited by the size of the array saved in RAM. With the current design, this maximum lies at an array of about 70 000 uint32_t values.

#### 2.2.3 Command line interface

A Command Line Interface (CLI) allows the user to operate the device through a serial console on their PC using a set of commands specified in the firmware. Communication between the PC and the microcontroller takes place over serial interface. The firmware is implemented as a loop which continuously calls a function that parses any data arriving through the serial port in search for user commands. If any existing command matches the user input, the corresponding function is executed and a feedback message is printed to inform the user of the successful input. If the input does not match any of the expected commands, the message “SIGULS: Unknown Command” is printed. The available commands are:

1. “SIGULS.Ping”: SIGULS responds to show it is functioning properly.
2. “SIGULS.Start”: SIGULS loads the data samples for signal generation into RAM and begins the output of the signal.
3. “SIGULS.Stop”: SIGULS stops the output of the signal.

#### 2.2.4 Generating the output signal

To output an analog signal, values need to be continuously sent to the DAC via SPI, and the DAC’s output update needs to be triggered with consistent timing.

The Load DAC Logic Input Pin ($\bar{\text{LDAC}}$) is used to update the output of the DAC to the last value written to it through the previously described SPI interface. This pin is controlled with a hardware PWM signal to ensure consistent timing. The same PWM signal routed to the $\bar{\text{LDAC}}$ pin and used to trigger the DAC update is also fed back to a General Purpose Input/Output (GPIO) pin on the microcontroller. This pin is configured to trigger an interrupt that looks up, prepares, and writes the next value to the DAC on the rising edge of the PWM signal. Using this method, DAC update frequencies (sampling rates) of 100 kHz could be achieved in a robust way. This was verified by looking at the output signal on an oscilloscope. Since the target is to generate excitation signals of up to 10 kHz in frequency, this sampling rate would allow for a resolution in time of 10 samples per period on such signals.

**Fig. 5:**
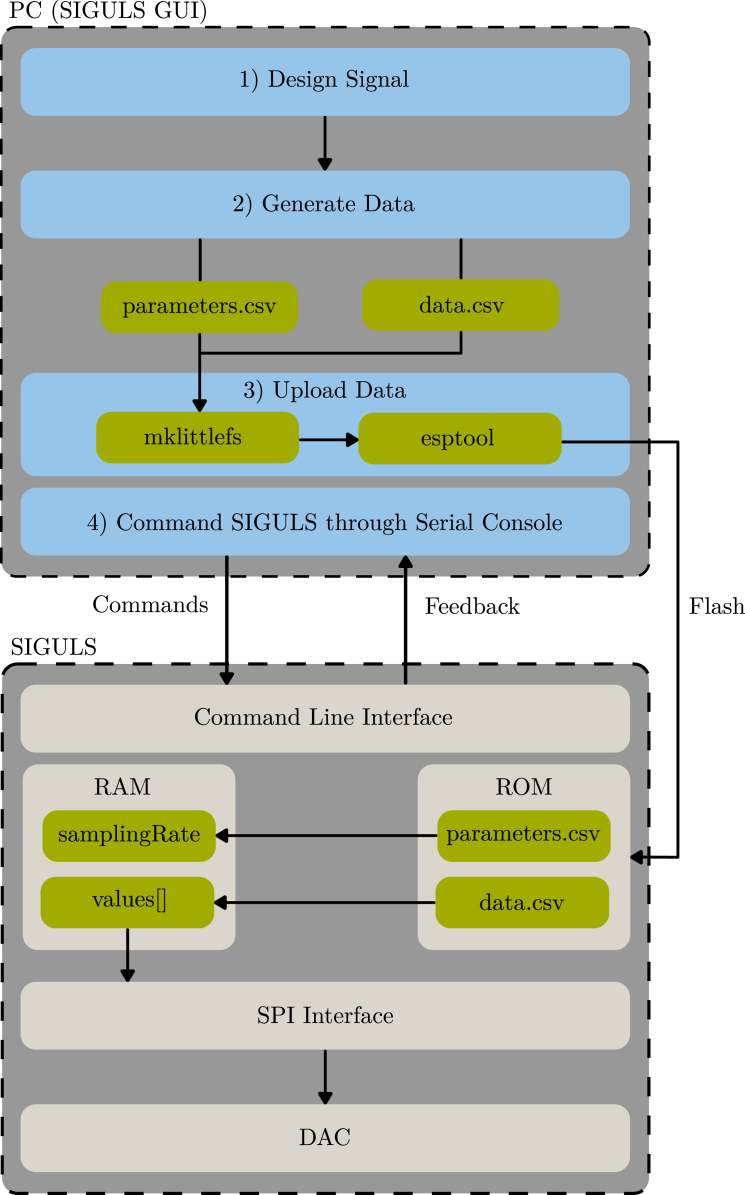
Simplified schematic of the steps from generating an array of values on the PC to outputting a signal from Siguls.

Fig. 5 summarizes the steps from generating an array of values on the PC to outputting a signal from SIGULS.

## 3 Design files summary

**Table:** 

**Design filename**	**File type**	**Open source license**	**Location of the file**
siguls_hardware.zip	KiCad project folder (ECAD files)	CC-BY 4.0	https://doi.org/10.5281/zenodo.17400147

jlcpcb_mfg.zip	JLCPCB manufacturing files	CC-BY 4.0	https://doi.org/10.5281/zenodo.17400147

siguls_firmware.zip	ESP32 source code (PlatformIO, Arduino Framework)	MIT	https://doi.org/10.5281/zenodo.17400147

siguls_gui.zip	Python code	MIT	https://doi.org/10.5281/zenodo.17400147

siguls_case.zip	STL file for 3D printing	CC-BY 4.0	https://doi.org/10.5281/zenodo.17400147

**siguls_hardware.zip** The KiCad project folder containing all of the electronic design files.

**jclpcb_mfg.zip** A folder containing all of the ECAD files needed to order the board from JLCPCB.

**siguls_firmware.zip** A folder containing the firmware to be flashed on the board.

**siguls_gui.zip** A folder containing all the python code required to run the SIGULS GUI.

**siguls_case.zip** The STL files of the 3D printed case for SIGULS.

## 4 Bill of materials summary

**Table:** 

Designator	Component	Number	Cost per unit - currency	Total cost - currency	Source of materials	Material type
PCB	Printed and partially assembled PCB	1	80€	80€	JLCPCB	Other

J1,J2	712-CONBNC002	2	1.83€	3.66€	712-CONBNC002 (Mouser)	Other
U2	AD5781BRUZ	1	46.94€	46.94€	584-AD5781BRUZ (Mouser)	Semiconductor
L3	SMDRI127-221MT	1	0.18€	0.18€	C10151 (JLCPCB)	Inductor
AE1	RFANT3216120A5T	1	0.06€	0.06€	C127629 (JLCPCB)	Antenna
L100	0402WGF0000TCE	1	0.01€	0.01€	C17168 (JLCPCB)	Jumper/0 Ω Link
U7	ICL7662CBA+T	1	1.31€	1.31€	C242528 (JLCPCB)	Semiconductor
U80	USBLC6-2P6	1	0.10€	0.10€	C2827693 (JLCPCB)	ESD protection
U90	TLV76733DRVR	1	0.21€	0.21€	C2848334 (JLCPCB)	LDO regulator
U8	MC34063AD	1	0.31€	0.31€	C2873086 (JLCPCB)	DC-DC converter
Y100	SX2B40.000F1210F30	1	0.08€	0.08€	C2901733 (JLCPCB)	Crystal
U1	ESP32-S3FN8	1	3.36€	3.36€	C2913196 (JLCPCB)	MCU/SoC
J80	GT-USB-7010ASV	1	0.07€	0.07€	C2988369 (JLCPCB)	USB connector

RESET, BOOT	TS-1101-C-W	2	0.04€	0.08€	C318938 (JLCPCB)	Tactile switch

U3	ADR445BRZ	1	8.85€	8.85€	C459149 (JLCPCB)	Voltage reference
F1	JK-nSMD050-30	1	0.03€	0.03€	C720075 (JLCPCB)	Fuse
D1	1N5819HW-7-F	1	0.02€	0.02€	C82544 (JLCPCB)	Diode
J200	U.FL-R-SMT-1(80)	1	0.09€	0.09€	C88374 (JLCPCB)	RF Connector

U4,U5, U6	AD8676ARZ	3	2.96€	8.88€	C99967 (JLCPCB)	Op-Amp

## 5 Build instructions

It is recommended to order the board almost fully assembled, except for the DAC and the two BNC connectors. To assemble these last few components, a soldering iron is required. Voltage checks should be performed to ensure that the board is in good working condition. Lastly, the firmware needs to be flashed onto the SIGULS device. SIGULS uses ±15V power lines, and appropriate safety precautions should be taken throughout the assembly and the usage of the device. As SIGULS is connected to powerful excitation devices like shakers during usage, precautions are also to be taken with respect to mechanical hazards.

### 5.1 Voltage checks

**Fig. 6:**
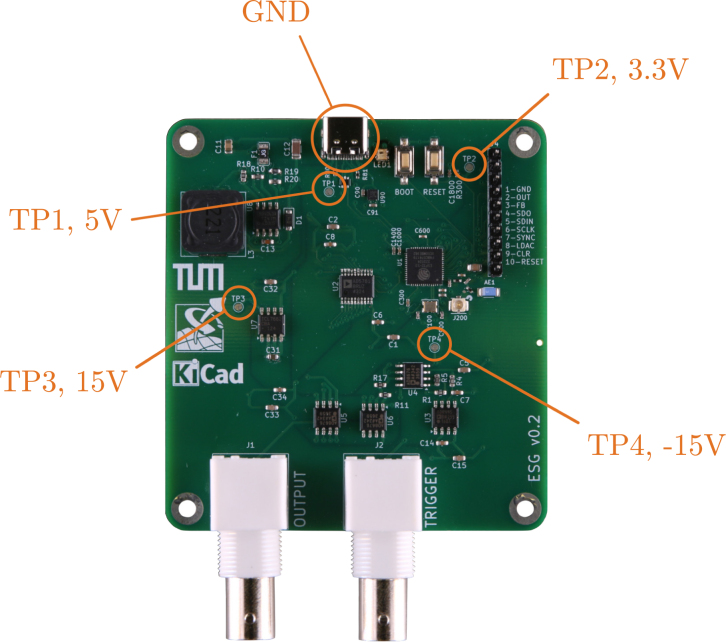
The SIGULS PCB with labeled test points.

To check all the voltage levels required by SIGULS using a multimeter, four test points are provided (see Fig. 6):

• TP1 → 5 V, if this voltage is not met, check that the USB-C connector on the board is working correctly.
• TP2 → 3.3 V, if this voltage is not met, check that the 3.3 V regulator on the board is working correctly.
• TP3 → 15 V, if this voltage is not met, check that the step-up converter on the board is working correctly.
• TP4 → -15 V, if this voltage is not met, check that the negative converter on the board is working correctly.

### 5.2 Soldering the missing components

**Fig. 7:**
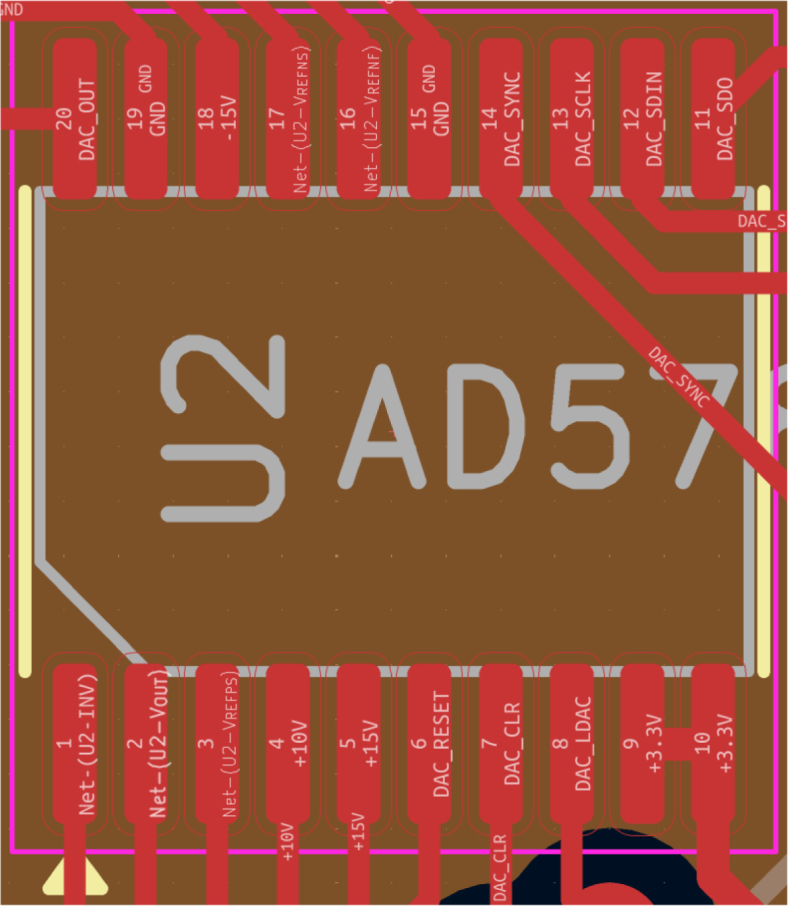
The footprint of the *AD5781* DAC from the KiCad PCB design. This figure should assist the soldering process by showing the orientation and pinout of the component.

Because the DAC is a particularly expensive component, it is recommended to order it separately and perform the voltage checks suggested previously before soldering it. The orientation of the DAC should be such that pin 1 (marked by the circle on the chip) ends up on the bottom left (see Fig. 7). As for the two BNC connectors, these are through-hole components as opposed to the rest of components, which are surface-mount. If the BNC connectors were assembled by the PCB manufacturer, this would require an additional assembly step, increasing the assembly cost. These should hence also be ordered separately and soldered on by hand. Note that, in the bill of materials, standard resistors and capacitors have been omitted.

### 5.3 Adding a case

The SIGULS device can optionally be placed in a case, for safety and decorative purposes. A 3D-printer is required for this step. The case can be 3D-printed from the files *Siguls_Case_Bottom* and *Siguls_Case_Top*. When slicing the part for printing, it is recommended to orient the two parts with their hollow sides (the side where the device is placed) pointing up to minimize the use of support material. Since the case is mostly decorative and does not protect the device from impacts, the infill can be chosen freely. The PCB can be attached to the bottom part of the case using M3 screws, which are screwed into the 3D-printed material. The top part of the case should fit snugly onto the bottom part.

### 5.4 Flashing the firmware

*PlatformIO* is used to flash the firmware onto SIGULS. The simplest way to use *PlatformIO* is through the *Visual Studio Code* (VSCode) extension. First, install and open VSCode.^1^ Next, open the “Extensions” tab, search for “PlatformIO IDE” and click “Install”. Go to “File”→“Open Folder” and select the project root (the directory containing the platformio.ini file). Connect SIGULS to the host PC using a USB-C cable. Once the project has been initialized, on the bottom toolbar, click “Build” followed by “Upload” to build and flash the firmware. It may be necessary to specify the serial port that the SIGULS device is connected to before uploading. If the board is being flashed for the first time, it may also be necessary to hold down the “BOOT” button while connecting the board, then unplug after flashing. SIGULS should now be ready to operate.

## 6 Operation instructions

### 6.1 Using the SIGULS GUI

A GUI is provided to offer a simple way for the user to design excitation signals of the most common types and generate them using the SIGULS device (see Fig. 8). The GUI can be installed using the pip Python package manager (pip install siguls-gui), and can be opened by typing “siguls-gui” in a terminal. The GUI has been tested to work on Linux (Ubuntu 24.04.3 LTS) and Windows 10 and 11. It uses Python version 3.12.3. The list of dependencies is as follows:

• PyQt5 5.15.11
• numpy 2.3.0
• esptool 4.8.1
• matplotlib 3.10.3
• pyExSi 0.43.3

To create and output a signal from SIGULS, take the following steps:Fig. 8Screenshots of the SIGULS GUI running on Linux (a) and Windows (b).Fig. 8

**(a):**
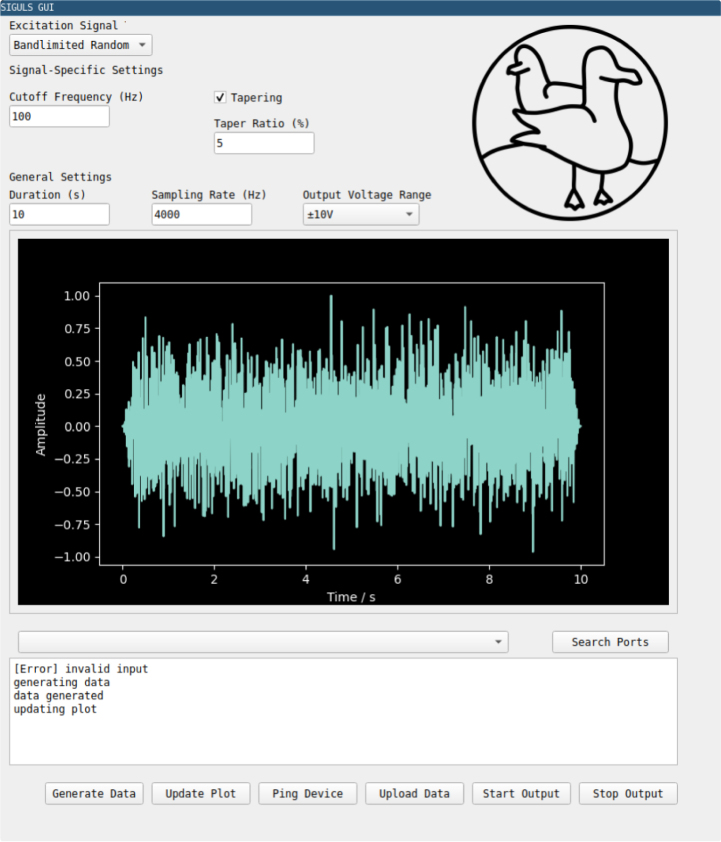
.

**(b):**
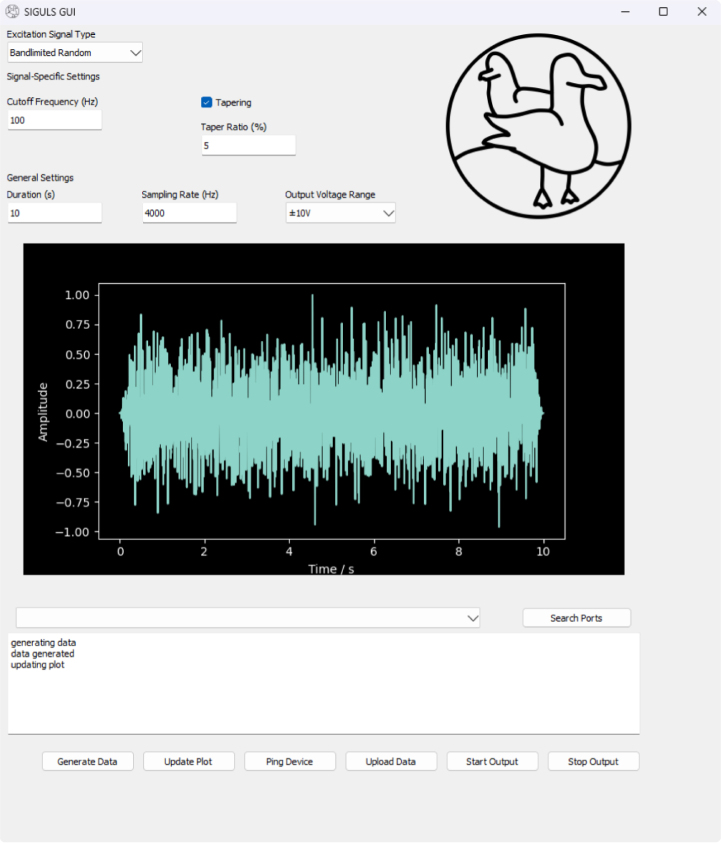
.

**Fig. 9:**
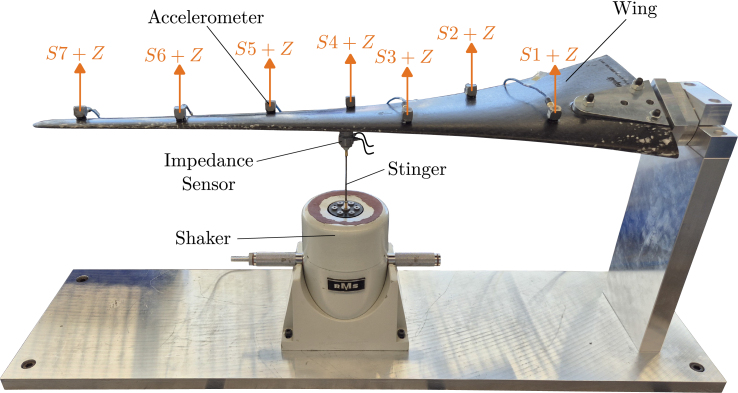
The experimental setup for the validation experiment. A carbon fiber wing is attached to a vibration-isolated table (not depicted). Excitation is carried out using a shaker which is controlled by a commercial DAQ with an integrated signal generator for one experiment and by the SIGULS device for the other. A stinger connects the shaker to the wing, and an impedance sensor between the tip of the stinger and the wing measures the excitation. The response of the wing is measured using seven tri-axial accelerometers. The positive z-axis of each accelerometer is indicated by a green arrow.

Fig. 10Frequency spectrum and time plot of the signal for excitation up to 100 Hz using **swept sine** excitation (a), and **bandlimited random** excitation (b). The signals are 16 s long, but only the first two seconds are shown. The swept sine excitation signals from both devices are nearly identical. As can be seen in the frequency spectrum plot for the bandlimited random excitation, the LMS achieves a steeper cut-off at 100 Hz when compared to the ESG. The ESG has a less noisy magnitude throughout the frequency range.Fig. 10

**(a):**
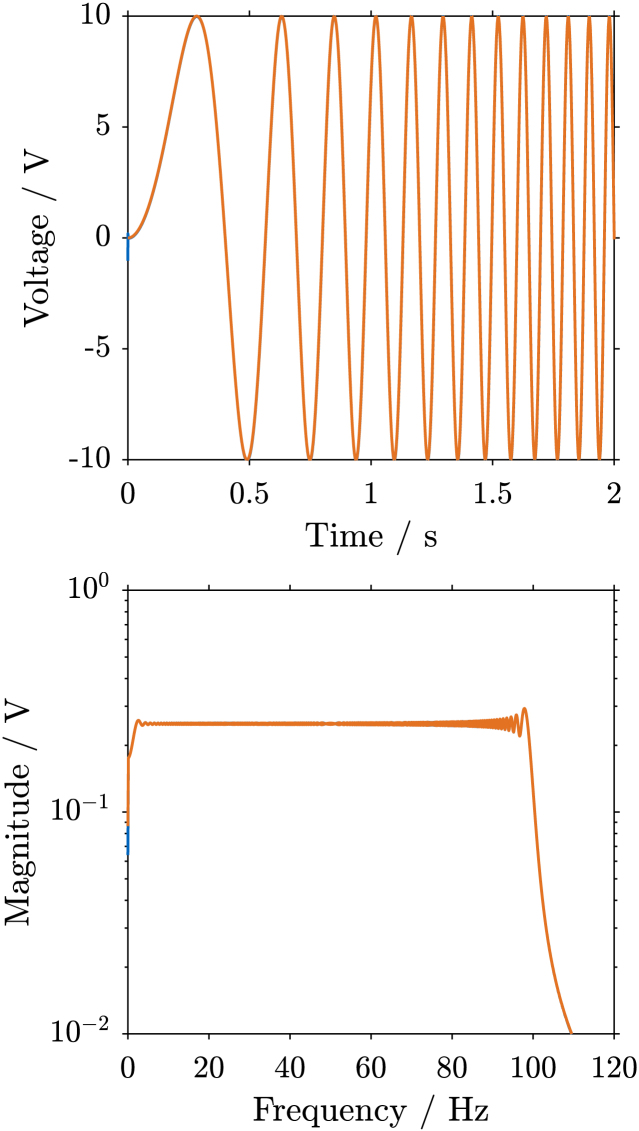
.

**(b):**
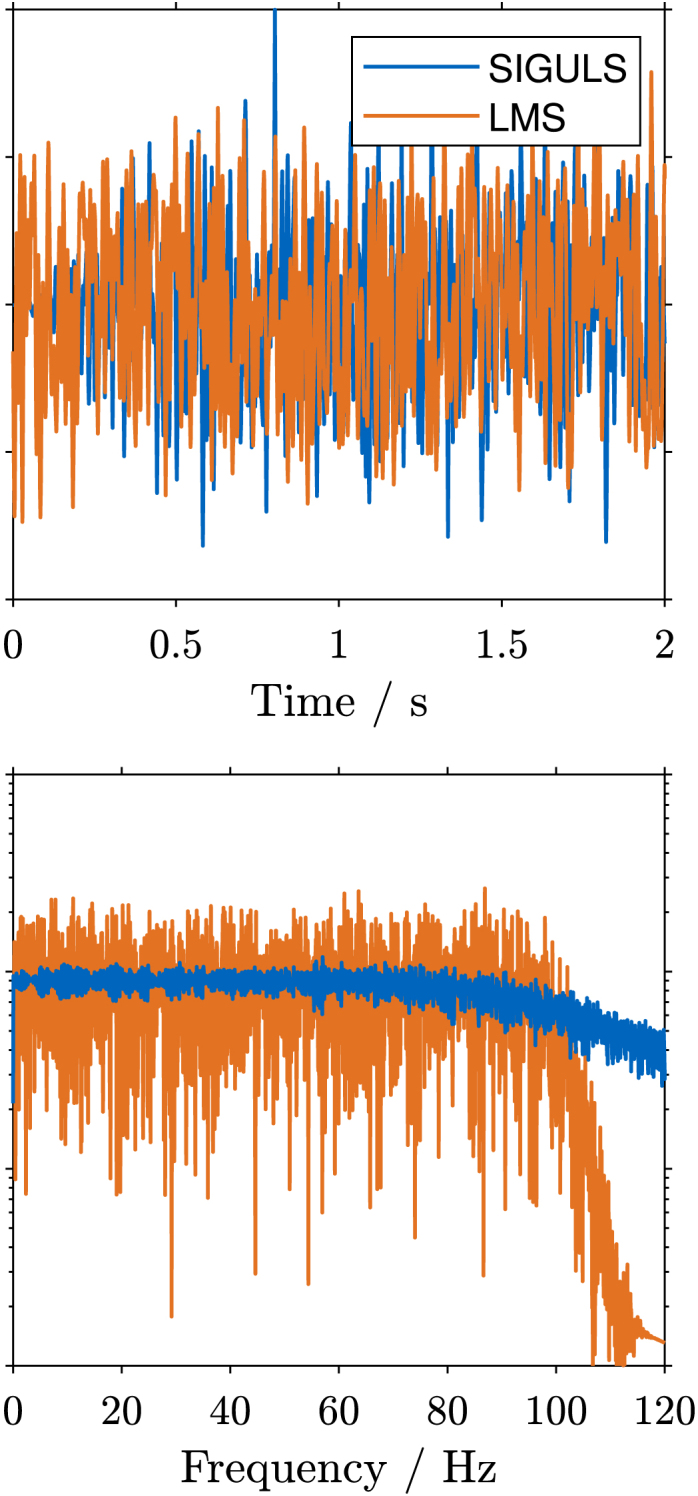
.Fig. 11Frequency Response Function (FRF) of sensor 7 in z-direction on the wing, including magnitude, phase, and coherence plots, for excitation up to 100 Hz using **swept sine** excitation (a), and **bandlimited random** excitation (b). The FRFs determined using SIGULS and the LMS look very similar for both methods of excitation. Increased noise is perceivable in SIGULS’s FRF for swept sine excitation around 50 Hz, correlating with a drop in coherence.Fig. 11

**(a):**
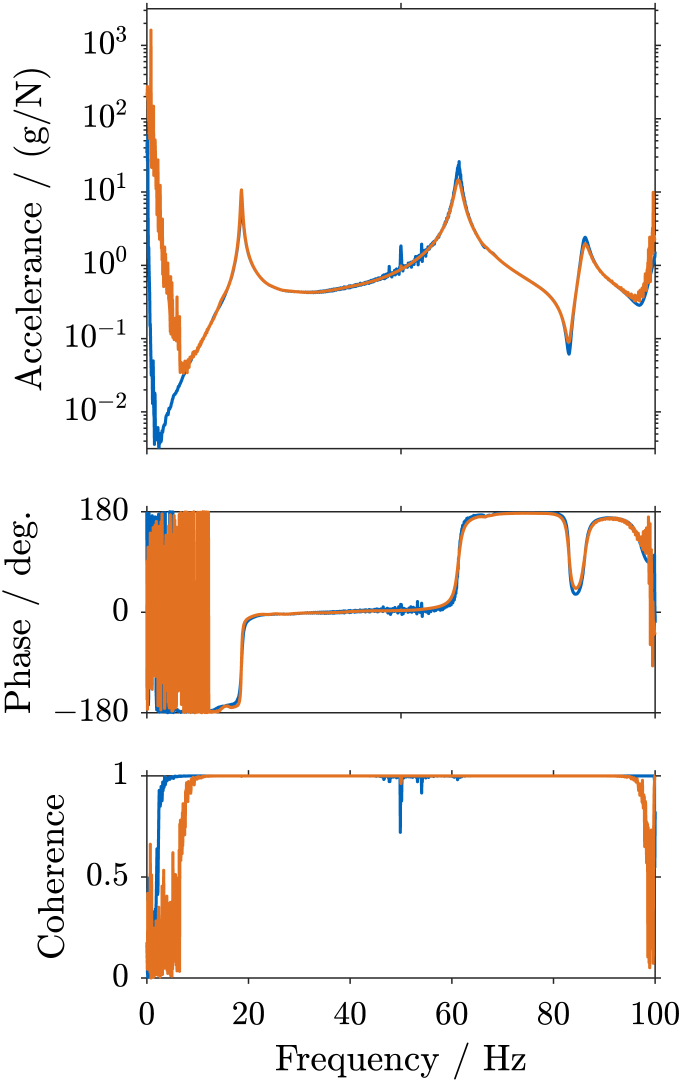
.

**(b):**
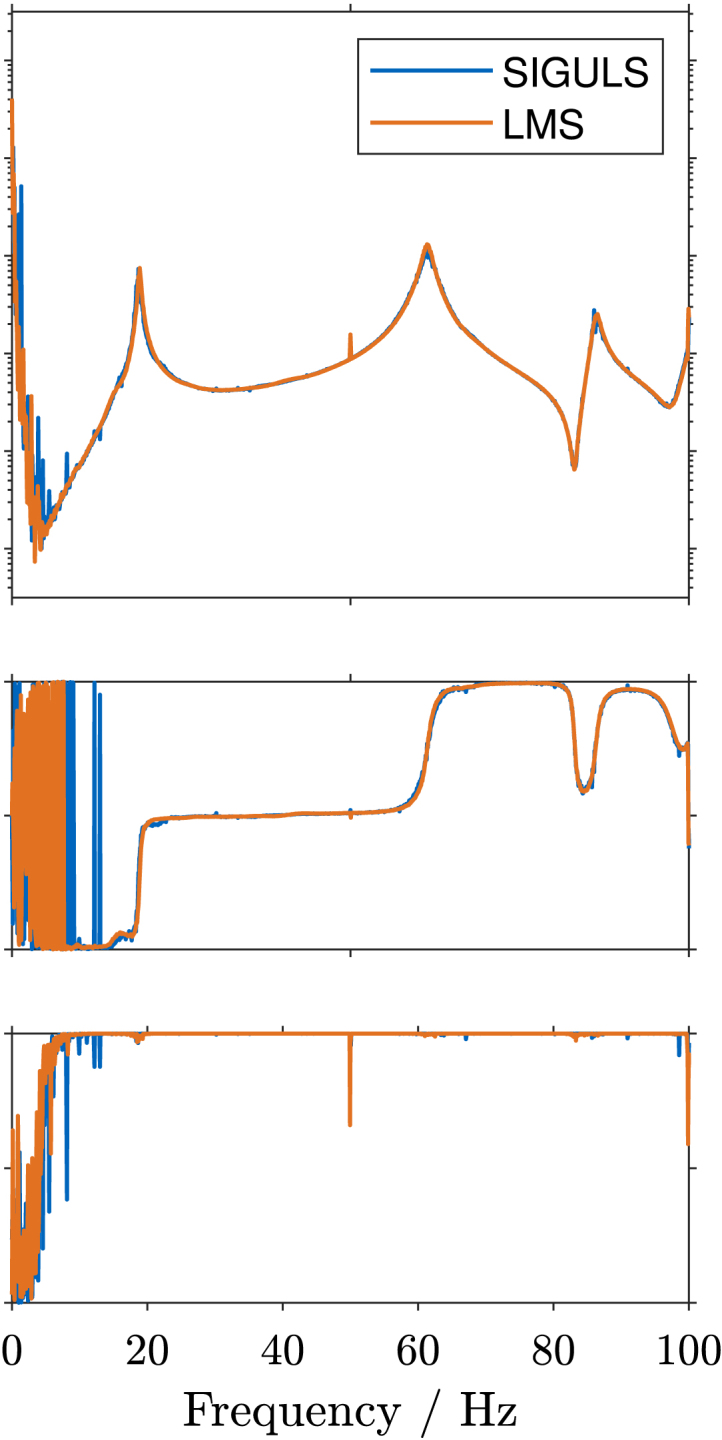
.

• Tune signal parameters and plot until the resulting signal is satisfactory.
• Connect output to power Amplifier
• Optionally connect trigger to DAQ trigger input
• Connect board to PC
• Click “Upload”
• Use the “Start Output” and “Stop Output” buttons as needed

### 6.2 Using alternative tools for signal design

• Export sample sequence to one-lined CSV file named “data.csv”
• Export sampling rate to a separate CSV file named “parameters.csv” as a single value
• Connect output to power Amplifier
• Optionally connect trigger to DAQ trigger input
• Connect board to PC
• Use the Siguls.uploadData() python function to flash data onto the board
• Use a Serial console of your choice (e.g., PuTTY) to send serial commands to ping the device (“SIGULS.Ping”), start the output (“SIGULS.Start”), or stop the output (“SIGULS.Stop”)

## 7 Validation and characterization

In this section, the presented excitation signal generator is put to use in structural dynamics tests in order to evaluate its performance and utility. Modal analysis is performed on a wing made out of carbon fiber reinforced plastics (CFRP) as the validation experiment. The *SIEMENS LMS SCADAS* data acquisition system is used to record all measurement signals for this experiment. Since this system can generate excitation signals of its own, the same experiments will be carried out with SIGULS’s and with the LMS’s excitation signals to compare SIGULS to a commercial product.

### 7.1 Experimental setup

An image of the wing with the surrounding elements of the test setup can be seen in Fig. 9. The wing is horizontally fixed at its wide end and excited using a shaker, placed roughly at the wing’s center. To measure the force input to the structure, an impedance sensor is placed between the stinger and the wing. The shaker is controlled by a signal from either the LMS SCADAS data acquisition system or SIGULS, both of which are amplified by a power amplifier. SIGULS’s random excitation signal in this experiment is a pseudo-random excitation signal. This means that, though the signal does not follow any deterministic function, it is the same random signal for every run of the experiment. Although SIGULS is capable of sampling rates up to 100 kHz a sampling rate of 4 kHz was chosen for this experiment, because it will allow us to output a 16 s long signal despite SIGULS’s signal length limit of 70 000 samples. The response of the structure at different locations is recorded using seven tri-axial accelerometers, where the sensors are numbered from the point at which the wing is fixed, with sensor 1 being the closest. To record the data from all experiments and to serve as a reference system for the generation of excitation signals, the LMS SCADAS data acquisition system is used. The Simcenter Testlab software was used for the recordings and the processing of the data to perform modal analysis. The experiment parameters for the tests on the wing test rig can be seen in Table 1.

**Table 1:** Experiment parameters for tests on the wing.

Measurement duration	16 s
Measurement bandwidth	4096 Hz
Spectral lines	66 536
Number of averages	5
Excitation signal (LMS)	Swept sine or band-limited random up to 100 Hz
Excitation signal (SIGULS)	Swept sine or band-limited pseudo-random up to 100 Hz
SIGULS sampling frequency	4 kHz

### 7.2 Results

The goal of the validation experiment is to perform modal analysis on the CFRP wing to identify the first three modes of the structure, using excitation signals generated by SIGULS and the LMS. This allows for a comparison between the results from both systems using the Modal Assurance Criterion (MAC). Excitation from 0 to 100 Hz is used, since previous experiments on this test rig show that the first three modes of the structure lie within this frequency range. The experiment in this chapter is carried out once using a random excitation signal and once using a swept sine for excitation.

The frequency spectra and time plots of the excitation signals are shown in Fig. 10. As can be seen for the swept sine excitation (Fig. 10(a)), the signal in the time domain, as well as the spectrum of the excitation signal closely matches the ones generated by the LMS. For the random excitation (Fig. 10(b)), it can be seen that the time domain shape is different, as one might expect for a random signal. The spectrum also shows some differences, mainly less noisy amplitudes for the SIGULS signal. Compared to the LMS, the drop-off above 100 Hz is less steep for SIGULS.

The Frequency Response Function (FRF) for Sensor 7 in z-direction (at the tip of the wing, in the vertical direction), determined with a swept sine excitation and with a bandlimited random excitation up to 100 Hz are shown in Fig. 11. For the most part, the FRFs determined using SIGULS and the LMS look very similar for both methods of excitation. Increased noise is perceivable in SIGULS’s FRF for swept sine excitation (Fig. 11(a)) around 50 Hz, correlating with a drop in coherence. The fact that this occurs at precisely 50 Hz, and the drop in coherence at the same frequency points towards a possible interference from the electrical grid, although no particular source of interference could be identified. For random excitation (Fig. 11(b)), this peak at 50 Hz accompanied by a drop-off in coherence is more pronounced for the LMS, indicating that this problem is not unique to the excitation signal from SIGULS. For swept sine excitation, SIGULS performs better than the reference at frequencies below 10 Hz and above 95 Hz, showing a more defined FRF with a higher coherence in this frequency range. The eigenfrequencies and damping ratios for each mode and each method of excitation are listed in Table 2. Eigenfrequencies and damping ratios characterize the measured system’s dynamics and are often identified using experimental modal analysis. Eigenfrequencies can be identified in the FRF by peaks at the same frequency across multiple points in the structure [1]. The sharpness of these peaks determines the damping ratio of the mode [1]. Modal parameters are identified using Simcenter Testlab with the *PolyMAX* algorithm [10].

The results of the comparison between the SIGULS experiments and the LMS experiments using the modal assurance criterion can be seen in (a), Fig. 12 for the swept sine, and in Fig. 12(b) for the random excitation. Ideally, the numbers on the diagonal would be very close or equal to 100 %, meaning that the modes identified from the data gathered using SIGULS and the LMS are identical. Since almost all values on the diagonal for random and swept sine excitation are above 99.9 %, the modes are highly self-correlated, meaning that there are only slight differences in the identification of modes when using the two systems for excitation. The values off the diagonal would ideally be close to zero, indicating that there is little correlation between different modes identified using the two excitation systems. Since all values are below 4 % we can be confident that modes 1–3 are truly distinct from one another [11].Fig. 12Modal Assurance Criterion (MAC) comparing the first three modes of the CFRP wing as identified by SIGULS and the LMS for **swept sine** excitation (a), and **bandlimited random** excitation (b). Ideally, the numbers on the diagonal would be very close or equal to 100 %. Since almost all values on the diagonal for random and swept sine excitation are above 99.9 %, the modes are highly self-correlated. The values off the diagonal would ideally be close to zero, indicating that there is little correlation between different modes identified using the two excitation systems. Since all values are below 4 % we can be confident that modes 1–3 are truly distinct from one another [11].Fig. 12

**(a):**
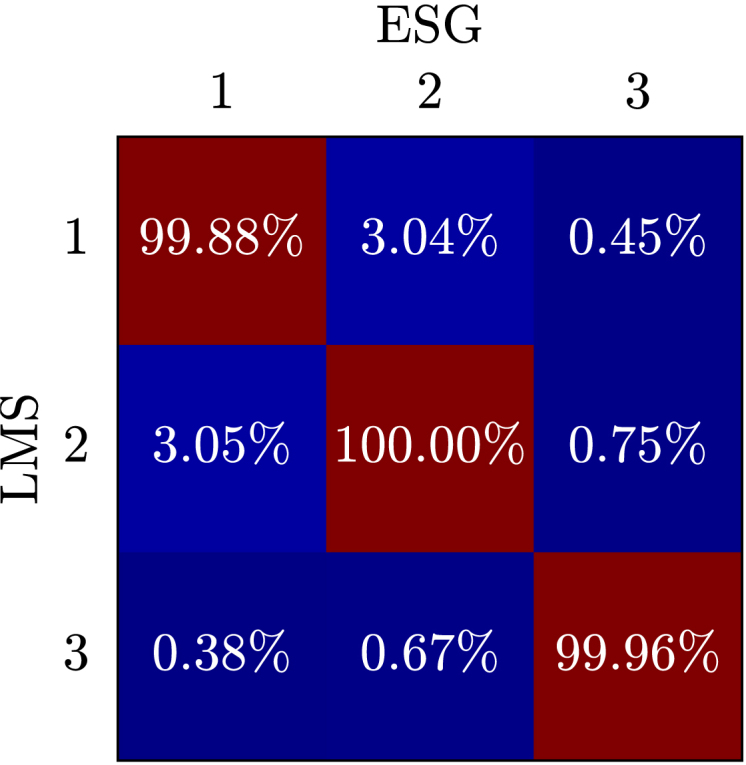
.

**(b):**
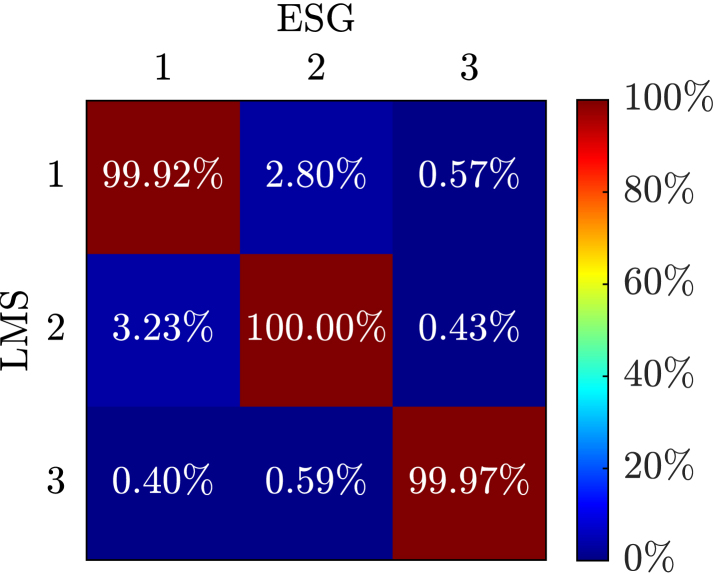
.

**Table 2:** Eigenfrequencies and damping ratios for different excitation methods.

Excitation method	Mode	Eigenfrequency (Hz)	Damping ratio (%)
LMS, swept sine	1	18.6	0.81
	2	61.2	1.09
	3	86.1	0.95

SIGULS, swept sine	1	18.5	1.47
	2	61.2	0.69
	3	86.0	0.75

LMS, bandlimited random	1	18.8	1.25
	2	61.4	1.20
	3	86.2	0.78

SIGULS, bandlimited random	1	18.6	1.50
	2	61.6	1.26
	3	86.1	0.86

In conclusion, the SIGULS device offers comparable performance to commercial alternatives in structural dynamics experiments, while being affordable and offering more flexibility for the design of excitation signals.

## CRediT authorship contribution statement

**Abelardo Pérez Paz:** Writing – review & editing, Writing – original draft, Visualization, Validation, Software, Methodology, Investigation, Formal analysis, Data curation, Conceptualization. **Oliver Maximilian Zobel:** Writing – review & editing, Validation, Methodology, Investigation, Formal analysis, Conceptualization. **Daniel J. Rixen:** Writing – review & editing, Supervision, Resources.

## Declaration of competing interest

The authors declare that they have no known competing financial interests or personal relationships that could have appeared to influence the work reported in this paper.

## Footnotes

1 https://code.visualstudio.com/.

## References

[1] Crystal Instruments (2016). https://www.crystalinstruments.com/basics-of-modal-testing-and-analysis.

[2] Horowitz Paul, Hill Winfield (2015). https://www.cambridge.org/us/universitypress/subjects/physics/electronics-physicists/art-electronics-3rd-edition?format=HB.

[3] Dumitriu Cosmin Dan, Pamfil Doru (2018). MATEC Web of Conferences.

[4] Brüel & Kjær (1988). https://www.bksv.com/es/knowledge/blog/vibration/structural-testing-part-one.

[5] (2010). https://www.analog.com/media/en/technical-documentation/data-sheets/ad5781.pdf.

[6] (2011). https://www.analog.com/media/en/reference-design-documentation/reference-designs/CN0177.pdf.

[7] (2024). https://www.espressif.com/sites/default/files/documentation/esp32-s3_datasheet_en.pdf.

[8] Zorman Aleš, Gorjup Domen, Slavič Janko (2020). https://pyexsi.readthedocs.io/en/latest/code.html.

[9] (2016). https://docs.espressif.com/projects/esp-iot-solution/en/latest/storage/file_system.html.

[10] Peeters B., Van der Auweraer H., Guillaume P., Leuridan J. (2004). The PolyMAX frequency-domain method: A new standard for modal parameter estimation?. Shock. Vib..

[11] Pastor Miroslav, Binda Michal, Harčarik Tomáš (2012). Modal assurance criterion. Procedia Eng..

